# Mastoparans: A Group of Multifunctional α-Helical Peptides With Promising Therapeutic Properties

**DOI:** 10.3389/fmolb.2022.824989

**Published:** 2022-06-24

**Authors:** Carlos José Correia de Santana, Osmindo Rodrigues Pires Júnior, Wagner Fontes, Mário Sérgio Palma, Mariana S. Castro

**Affiliations:** ^1^ Laboratory of Toxinology, Department of Physiological Sciences, Institute of Biological Sciences, University of Brasília, Brasília, Brazil; ^2^ Laboratory of Protein Chemistry and Biochemistry, Department of Cell Biology, Institute of Biological Sciences, University of Brasília, Brasília, Brazil; ^3^ Department of Basic and Applied Biology, Institute of Biosciences of Rio Claro, São Paulo State University, UNESP, Rio Claro, Brazil

**Keywords:** social and solitary wasps, venom, linear cationic α-helical peptide, mastoparans, mast cell degranulation, antimicrobial peptide

## Abstract

Biologically active peptides have been attracting increasing attention, whether to improve the understanding of their mechanisms of action or in the search for new therapeutic drugs. Wasp venoms have been explored as a remarkable source for these molecules. In this review, the main findings on the group of wasp linear cationic α-helical peptides called mastoparans were discussed. These compounds have a wide variety of biological effects, including mast cell degranulation, activation of protein G, phospholipase A_2_, C, and D activation, serotonin and insulin release, and antimicrobial, hemolytic, and anticancer activities, which could lead to the development of new therapeutic agents.

## Introduction

Mastoparans are small peptides (commonly tetradecapeptides with an amidated C-terminal) that were originally described for promoting degranulation and the release of histamine in mast cells. This group of peptides is found in the venom of several species of social and solitary wasps (Hirai et al., 1979b; Konno et al., 2016; Lee et al., 2016; dos Santos Cabrera et al., 2019; Abd El-Wahed et al., 2021). Several studies have demonstrated that mastoparans have a wide variety of biological effects, including activation of protein G (Higashijima et al., 1988), phospholipase A_2_, C, and D activation (Cho et al., 1995; Himbergen et al., 1999), serotonin and insulin release (Ozaki et al., 1990), antimicrobial, hemolytic, and anticancer activities (Park et al., 1995; Yamada et al., 2005; Rangel et al., 2011; Wang et al., 2014; de Azevedo et al., 2015), and other properties. In plants, mastoparans were used to promote the intracellular Ca^+2^ increase to regulate cell-to-cell communication (Tucker and Boss, 1996).

**FIGURE 1:**
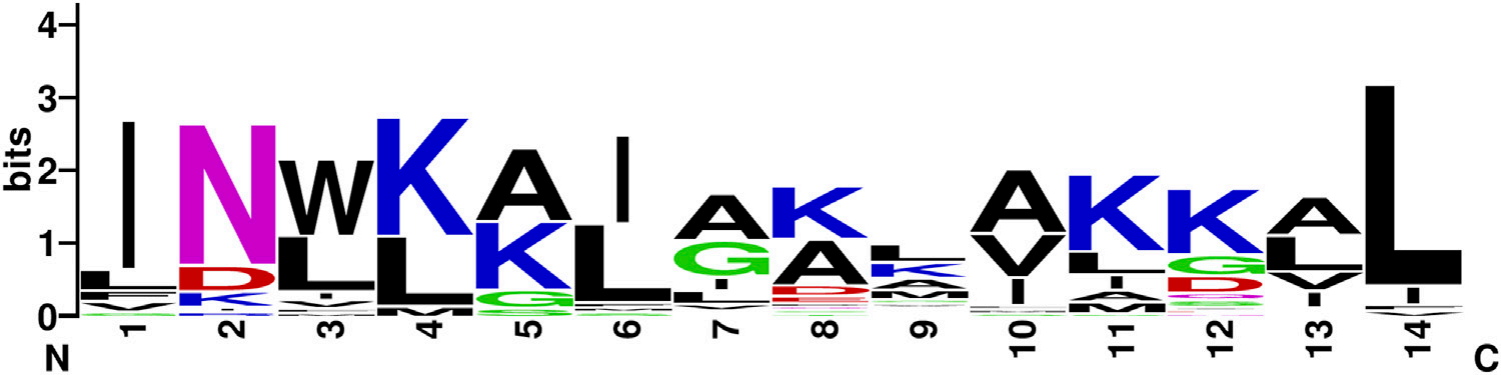
Consensus sequence of mastoparans (with 14 amino acid residues) using the WebLogo generator, a graphical representation of the patterns within a multiple sequence alignment, in which the overall height of each stack indicates the sequence conservation at that position (measured in bits), whereas the height of the symbols within the stack reflects the relative frequency of the corresponding amino acid at that position (Crooks, 2004).

The consensus sequence of mastoparans using a WebLogo generator is indicated in Figure 1. Most mastoparans have a length of 14 amino acid residues, (with the exceptions of mastoparan-VT7, mastoparan-like peptide-12b, and mastoparan-VT2 containing 13 amino acid residues; mastoparan-V1 and–V2 containing 15 amino acid residues; and dominulin-A, dominulin-B, and PMM with 17 amino acid residues), and a net positive charge with an amidated C-terminus. They also have a high content of hydrophobic residues (Ile, Leu, Ala, Trp, and Val), which are commonly placed at positions 1, 3, 6, 10, 13, and 14, while positions 4, 11, and 12 are usually positively charged residues (Figure 1).

**FIGURE 2:**
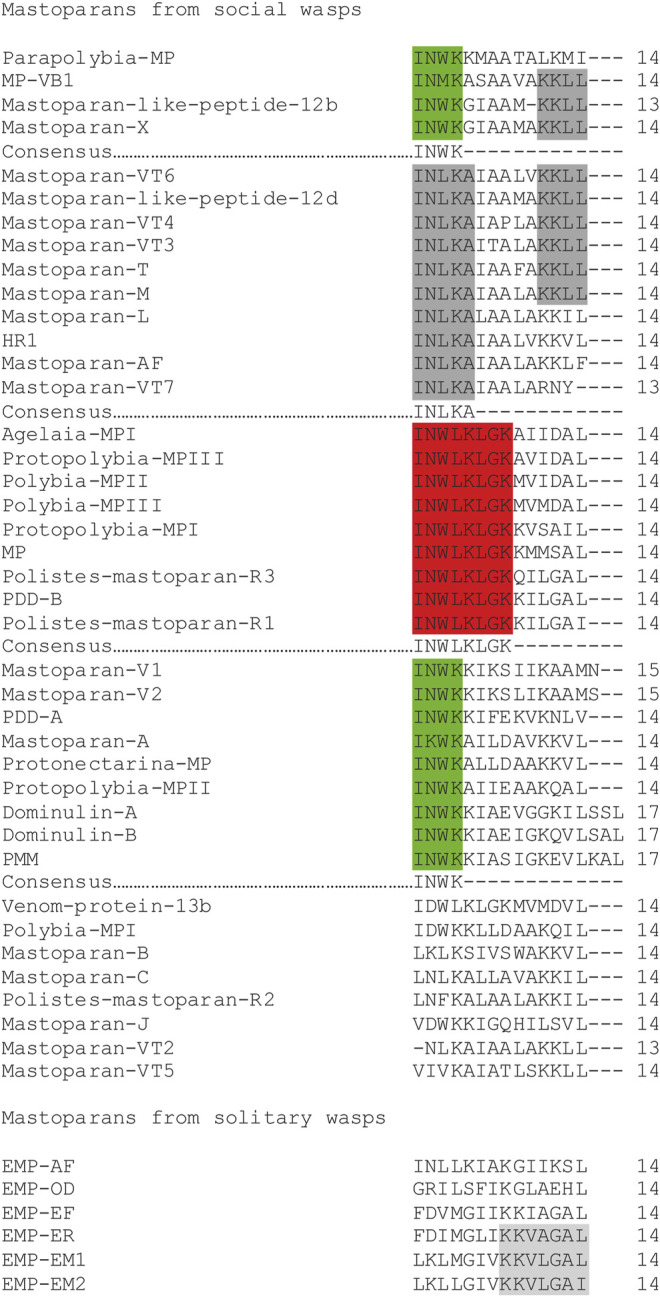
Amino acid sequence alignment of mastoparans. The sequence motifs are highlighted. Gaps have been inserted to improve visualization. Some manual changes have been made to improve the visualization and understanding.

There are 40 mastoparan sequences described in the venoms of social wasps and six amongst the solitary wasps. The primary structures of mastoparans are more conserved in social than those in solitary wasps, and they are characterized by the sequence motifs in the N-terminal region of the molecules: INWK … ; INLKA … ; and INWLKLGK … ; these sequence features were not observed amongst the solitary wasps (Figure 2).

The natural mastoparans usually present from one to four lysine residues in their sequences, creating variability of positive charges localized at different positions along the peptide molecule (from +1 to +5), creating a series of molecules with different charged surface densities. The aforementioned observation seems to be an evolutive strategy that creates a series of peptides with affinities for different cell types. According to Figure 2, the more common positions of lysine residues amongst the social wasps are:

- In total, two lysine residues occur at the positions: 4–5, 4–11, and 5–8;
- In total, three lysine residues occur at the positions: 2–8–9, 3–10–11, 4–5–11, 4–5–12, 4–11–12 (the most common one), and 5–8–9;
- In total, four lysine residues occur at the positions: 2–4–11–12, 4–5–7–11, 4–5–9–11, and 4–5–11–15.

The mastoparans from the venoms of solitary wasps also present a different number of lysine residues along the peptide sequence, in which the most representatives present different patterns of distribution of this residue, from those discussed earlier for the social wasps. Therefore, the patterns of lysine positioning along the sequence for the solitary wasp mastoparans are the following:

- A total of one lysine residue occurs at the position: 8;
- A total of two lysine residues occur at the positions: 8–9;
- A total of three lysine residues occur at the positions: 2–8–9 and 5–8–12.

Table 1 shows the sequences of the known natural mastoparans from venoms of social and solitary wasps with their main biological properties.

**TABLE 1:** Natural mastoparans from social and solitary wasps and their biological activities.

Social wasps
Name	Primary structure	Species	Biological activity	Reference
Agelaia-MPI	INWLKLGKAIIDAL-NH_2_	*Agelaia pallipes*	Mast cell degranulation, antimicrobial, hemolytic, antinociceptive, and insulin secretion in mice pancreatic islets	Mendes et al. (2004), Baptista-Saidemberg et al. (2012), Gonçalves et al. (2016), das Neves et al. (2019)
		*Parachartergus fraternus*		
Dominulin-A	INWKKIAEVGGKILSSL-NH_2_	*Polistes dominulus*	Antimicrobial	Turillazzi et al. (2006)
Dominulin-B	INWKKIAEIGKQVLSAL-NH_2_	*Polistes dominulus*	Antimicrobial	Turillazzi et al. (2006)
HR1	INLKAIAALVKKVL-NH_2_	*Vespa orientalis*	Mast cell degranulation, antimicrobial, hemolytic, and anticancer	Nakao et al. (2011), da Silva et al. (2018)
Mastoparan-A	KWKAILDAVKKVL-NH_2_	*Vespa analis*	Antimicrobial and anticancer	Yoon et al. (2015)
Mastoparan-AF	INLKAIAALAKKLF-NH_2_	*Vespa affinis*	Mast cell degranulation, antimicrobial, and hemolytic	Lin et al. (2012)
Mastoparan-B	LKLKSIVSWAKKVL-NH_2_	*Vespa basalis*	Mast cell degranulation, antimicrobial, hemolytic, edematogenic, and hypotensive	Ho and Hwang, (1991), Ho et al. (1994), Park et al. (1995), Ho et al. (2001)
Mastoparan-C	LNLKALLAVAKKIL-NH_2_	*Vespa crabro*	Mast cell degranulation, antimicrobial, hemolytic, and anticancer	Yoon et al. (2015), Chen et al. (2018), Hassan et al. (2021)
Mastoparan-J	VDWKKIGQHILSVL-NH_2_	*Polistes jokahamae* (*Polistes jadwigae*)	Mast cell degranulation and anticancer	Hirai et al. (1980), Raynor et al. (1992)
Mastoparan-L	INLKALAALAKKIL-NH_2_	*Vespula lewisii* (Vespula flaviceps lewisii)	Mast cell degranulation, antimicrobial, chemotactic for PMNL^a^, anticancer, and anxiolytic	Katsu et al. (1990), Raynor et al. (1992), Halling, (1996), Kamath et al. (2001), Vila-Farres et al. (2012), de Azevedo et al. (2015), Hilchie et al. (2016), Arifuzzaman et al. (2019), Silva et al. (2020a)
Mastoparan-M Mastoparan-VT1/Mastoparan-like peptide 12c	INLKAIAALAKKLL-NH_2_	*Vespa magnifica*	Mast cell degranulation and antimicrobial	Lin et al. (2011), Yang et al. (2013b)
		*Vespa tropica*		
		*Vespa mandarinia*		
Mastoparan-T/mastoparan-D	INLKAIAAFAKKLL-NH_2_	*Vespa tropica* (*Sphex tropica*)	Mast cell degranulation and antimicrobial	Nakajima, (1986), Lin et al. (2011)
		*V. ducalis*		
Mastoparan-V1	INWKKIKSIIKAAMN	*Vespula vulgaris*	Mast cell degranulation and antimicrobial	King et al. (2003), Kim et al. (2016)
Mastoparan-V2	INWKKIKSLIKAAMS-NH_2_	*Vespula vulgaris*	Antimicrobial^b^ and chemotactic for PMNL^a^	King et al. (2003)
Mastoparan-VT2	NLKAIAALAKKLL	*Vespa tropica*	Antimicrobial	Yang et al. (2013b)
Mastoparan-VT3	INLKAITALAKKLL	*Vespa tropica*	Antimicrobial	Yang et al. (2013b)
Mastoparan-VT4	INLKAIAPLAKKLL	*Vespa tropica*	Antimicrobial	Yang et al. (2013b)
Mastoparan-VT5	VIVKAIATLSKKLL	*Vespa tropica*	Antimicrobial	Yang et al. (2013b)
Mastoparan-VT6	INLKAIAALVKKLL	*Vespa tropica*	Antimicrobial	Yang et al. (2013b)
Mastoparan-VT7	INLKAIAALARNY	*Vespa tropica*	Antimicrobial	Yang et al. (2013b)
Mastoparan-X/mastoparan-V/mastoparan-like peptide 12a	INWKGIAAMAKKLL-NH_2_	*Vespa magnifica*	Mast cell degranulation, antimicrobial, and anticancer	Hirai et al. (1979a), Raynor et al. (1992), Xu et al. (2006b), da Silva et al. (2018)
		*Vespa xanthoptera* (*V. simillima xanthoptera*)		
		*V. velutina*		
Mastoparan-like peptide 12b	INWKGIAAMKKLL-NH_2_	*Vespa magnifica*	Mast cell degranulation and antimicrobial	Xu et al. (2006b)
Mastoparan-like peptide 12d	INLKAIAAMAKKLL-NH_2_	*Vespa magnifica*	Mast cell degranulation and antimicrobial	Xu et al. (2006b)
MP	INWLKLGKKMMSAL-NH_2_	*Mischocyttarus phthisicus*	Mast cell degranulation and antimicrobial	Čeřovský et al. (2008)
MP-VB1	INMKASAAVAKKLL-NH_2_	*Vespa bicolor*	Mast cell degranulation and antimicrobial	Chen et al. (2008)
Parapolybia-MP	INWKKMAATALKMI-NH_2_	*Parapolybia indica*	Antimicrobial and hemolytic	de Souza et al. (2011)
PDD-A	INWKKIFEKVKNLV-NH_2_	*Polistes dorsalis*	Mast cell degranulation and antimicrobial	de Souza et al. (2011)
PDD-B	INWLKLGKKILGAL-NH_2_	*Polistes dorsalis*	Mast cell degranulation, antimicrobial, and hemolytic	Ho et al. (2001), de Souza et al. (2011)
PMM	INWKKIASIGKEVLKAL-NH_2_	*Polistes major*	Mast cell degranulation and antimicrobial	de Souza et al. (2011)
Polistes-mastoparan-R1	INWLKLGKKILGAI-NH_2_	*Polistes rothneyi iwatai*	Mast cell degranulation, antimicrobial, and hemolytic	Murata et al. (2006)
Polistes-mastoparan-R2	LNFKALAALAKKIL-NH_2_	*Polistes rothneyi iwatai*	Mast cell degranulation and hemolytic	Murata et al. (2006)
Polistes-mastoparan-R3	INWLKLGKQILGAL-NH_2_	*Polistes rothneyi iwatai*	Mast cell degranulation and hemolytic	Murata et al. (2006)
Polybia-MPI	IDWKKLLDAAKQIL-NH_2_	*Polybia paulista*	Mast cell degranulation, antimicrobial, anticancer, and chemotactic for PMNL^a^	de Souza et al. (2005), Wang et al. (2008, 2009), dos Santos Cabrera et al. (2012), da Silva et al. (2018), Li et al. (2019)
Polybia-MPII/venom protein 13a	INWLKLGKMVIDAL-NH_2_	*Polybia paulista*	Mast cell degranulation, antimicrobial, hemolytic, chemotactic for PMNL^a^, inflammatory, myotoxic, neurotoxic, and apoptotic	Rocha et al. (2007, 2008), Silva et al. (2017), das Neves et al. (2019)
		*Pseudopolybia vespiceps testacea*		
Venom protein 13b	IDWLKLGKMVMDVL-NH_2_	*Polybia paulista*	Mast cell degranulation, hemolytic, chemotactic for PMNL^a^, and trypanocidal	dos Santos Cabrera et al. (2004), Vinhote et al. (2017)
Polybia-MPIII	INWLKLGKMVMDAL-NH_2_	*Polybia paulista*	Mast cell degranulation, antimicrobial, hemolytic, and chemotactic for PMNL^a^	de Souza et al. (2005), de Souza et al. (2009)
Protonectarina-MP	INWKALLDAAKKVL-NH_2_	*Protonectarina sylveirae*	Mast cell degranulation and antimicrobial^b^	Dohtsu et al. (1993)
Protopolybia-MPI	INWLKLGKKVSAIL-NH_2_	*Protopolybia exigua*	Mast cell degranulation and hemolytic	Mendes et al. (2005)
Protopolybia-MPII	INWKAIIEAAKQAL-NH_2_	*Protopolybia exigua*	Mast cell degranulation	Mendes et al. (2005)
Protopolybia-MPIII	INWLKLGKAVIDAL-NH_2_	*Protopolybia exigua*	Mast cell degranulation	Mendes et al. (2005)

Solitary wasps
Name	Primary structure	Species	Biological activity	Reference
EMP-AF	INLLKIAKGIIKSL-NH_2_	*Anterhynchium flavomarginatum micado*	Mast cell degranulation, hemolytic, and antimicrobial	Konno et al. (2000)
EMP-EF	FDVMGIIKKIAGAL-NH_2_	*Eumenes fraterculus*	Mast cell degranulation, antimicrobial, and leishmanicidal	Rangel et al. (2011)
EMP-EM1	LKLMGIVKKVLGAL-NH_2_	*Eumenes micado*	Mast cell degranulation, antimicrobial, and leishmanicidal	Konno et al. (2019)
EMP-EM2	LKLLGIVKKVLGAI-NH_2_	*Eumenes micado*	Mast cell degranulation, antimicrobial, and leishmanicidal	Konno et al. (2019)
EMP-ER	FDIMGLIKKVAGAL-NH_2_	*Eumenes rubrofemoratus*	Mast cell degranulation, antimicrobial, and leishmanicidal	Rangel et al. (2011)
EMP-OD	GRILSFIKGLAEHL-NH_2_	*Orancistrocerus drewseni*	Antifungal and hemolytic	Murata et al. (2009), Baek and Lee, (2010)

a PMNL, polymorphonucleated leukocytes.

b Manual assertion inferred from sequence similarity according to UniprotKB.

**FIGURE 3:**
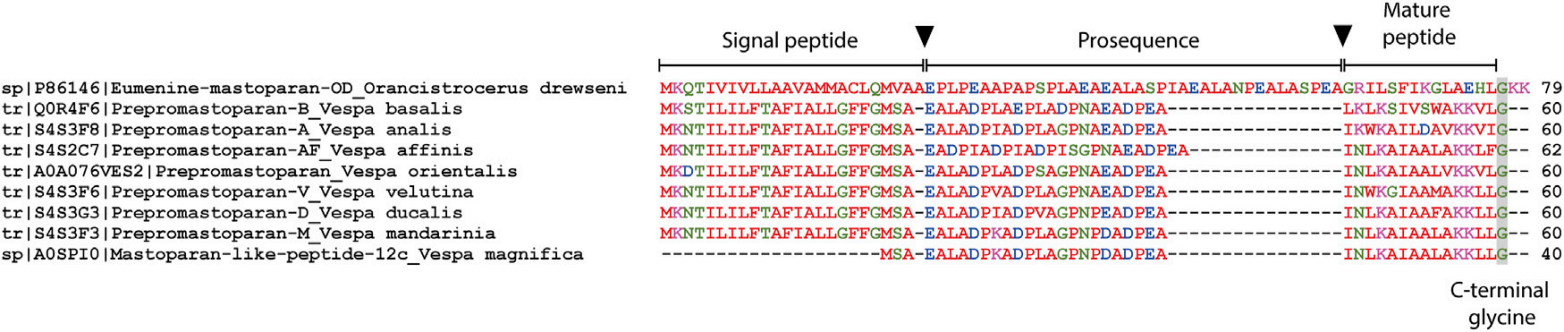
Amino acid sequence alignment of the translated mastoparan precursors obtained from UniprotKB. Arrows indicate signal peptide and prosequence removal points. The C-terminal glycine indicates the site of amidation of the mature peptide. Gaps have been inserted to improve visualization.

Despite considerable knowledge about the structure and biological activities of mastoparans, little is known about the biosynthesis of these molecules. In species of the genus *Vespa*, the precursors of mastoparans obtained from cDNA consist of a pre-pro-peptide containing mostly 60 amino acids. In relation to the other genera, only EMP-OD from the solitary wasp *Orancistrocerus drewseni* had its precursor investigated, and this molecule presents a significantly longer sequence in relation to the social wasps (Figure 3). A common feature of these molecules is the presence of an anionic sequence composed of 11 or 12 dipeptides for *Vespa* species or 19 dipeptides for *Orancistrocerus drewseni drewseni* mostly terminated in proline or alanine. These prosequence repeats are the targets for cleavage by proteases such as dipeptidyl peptidase IV, also found in the venom gland of *V. basalis* (Lee et al., 2007), whereas glycine at the C-terminal end is amidated by the action of enzymes such as peptidylglycine α-hydroxylating monooxygenase and peptidyl-α-hydroxyglycine α-amidating lyase (Lee et al., 2007).

The structural features described earlier will determine the type of cell which is the target of each toxin, and consequently the pharmacological effects. These peptides induce secretion of histamine from mast cells, serotonin from platelets, catecholamines from chromaffin cells, and prolactin from the anterior pituitary gland (Higashijima et al., 1988; Moreno and Giralt, 2015). In neutrophils, the mastoparans stimulate the reorganization of the cytoskeleton, production of the phosphatidylinositol 3,4,5-trisphosphate, up-regulated secretion of the complement receptor type 3, and superoxide anion formation (Norgauer et al., 1992).

**FIGURE 4:**
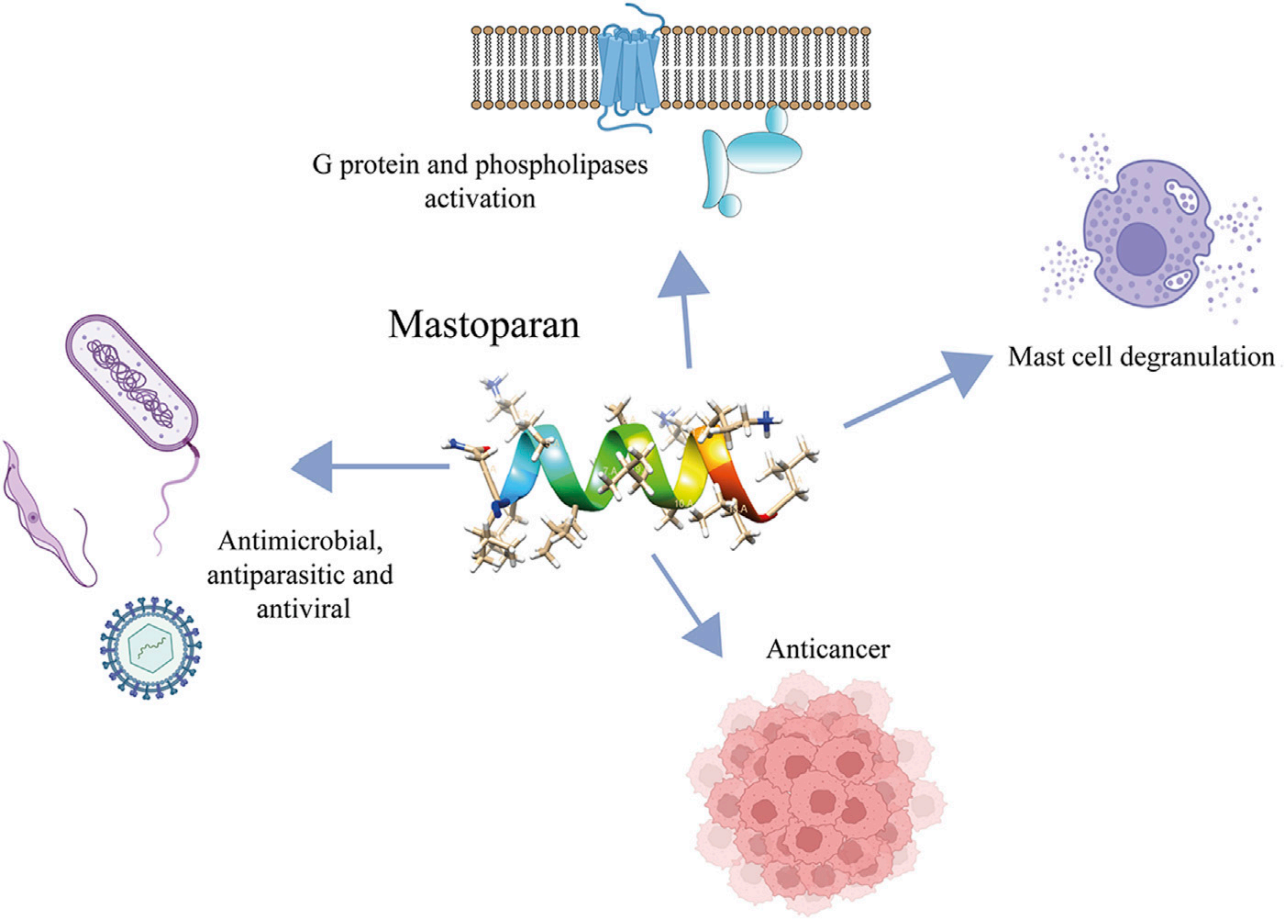
Main biological activities exhibited by mastoparans.

We present an overview of mastoparans and their analogs including their biological effects (Figure 4), mechanisms of action, and selectivity, which could lead to the development of new therapeutic agents.

## Mast Cell Degranulation

Mast cells are effector cells that act on the immune system and are associated with helper T cells and IgE, participating in inflammatory responses. They appear to act as sentinels in serous cavities and epithelial surfaces as well as near blood vessels, nerves, and glands. In addition, these cells can act in response to pathogens and their products, participating in innate and acquired immunity (Galli et al., 1999; Galli et al., 2005).

**FIGURE 5:**
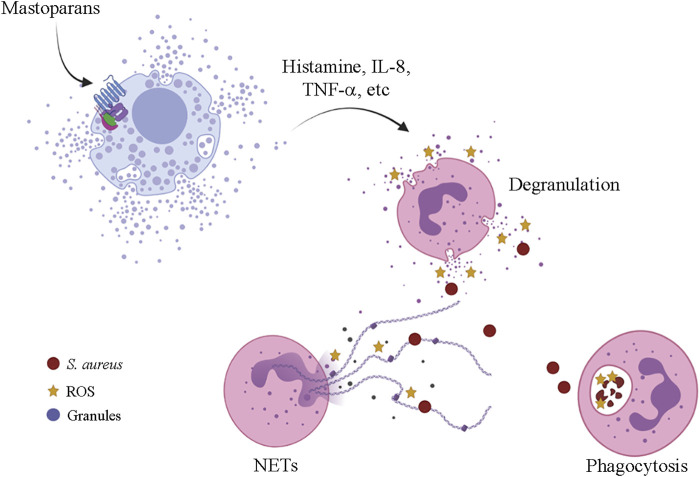
Mrgprb2-mediated mast cell activation by mastoparans leading to neutrophil recruitment and *S. aureus* clearance in a mouse dermonecrotic-infected lesion. ROS—reactive oxygen species; NETs—neutrophil extracellular traps.

Mast cell degranulation releasing mediators, such as histamine and serotonin, represent the main biological activity of the mastoparans and mastoparan-like peptides and this activity is responsible to collectively name this group of molecules (Hirai et al., 1979b; Moreno and Giralt, 2015). These molecules, isolated or associated with phospholipase A_2_, characterize the main symptoms of wasp envenomation, such as inflammation, pain, edema, and others (Moreno and Giralt, 2015). The mechanism of action that promotes the degranulation of mast cells is not yet fully elucidated but apparently appears to be related to the interaction with G proteins and to the lytic capacity of these peptides (Lorenz et al., 1998). The neuropeptide substance P (SP), that displayed similar activities to mastoparans, causes the activation of mast cells and it appears to be independent of receptors, interacting directly with the plasma membrane and stimulating the G proteins. Previous treatment with pertussis toxin and GDPbS blocked the degranulation in mast cells when in contact to the intracellularly applied SP (Lorenz et al., 1998). Neuraminidase treatment desensitized the mast cells to mastoparans, implying that its interaction to the target was facilitated by the negative charges of sialic acid residues (Mousli et al., 1990). McNeil et al. reported that mouse mast cells were activated (*in vitro* and *in vivo*) *via* a single receptor named Mrgprb2, an orthologous of human G-protein-coupled receptor MRGPRX2 (Mas-related G protein-coupled receptor member X2) (McNeil et al., 2015). Later, it was shown that MRGPR-mediated activation of connective tissue mast cells (CTMCs) by mastoparan-L resulted in neutrophil recruitment and accelerated the clearance of *Staphylococcus aureus* in a mouse dermonecrotic infection model *in vivo* (Figure 5). In addition, mice treated with mastoparan-L showed a reduction in the lesion when submitted to reinfection and suggesting an improvement of the adaptive immune response by mast cell modulation (Arifuzzaman et al., 2019).

*Protopolybia* mastoparans (MP-I, -II, and-III), isolated from *Protopolybia exigua*, displayed different levels of mast cell degranulation and hemolysis (Mendes et al., 2005). In the sub lethal concentration was observed that the Protopolybia MP-III peptide interacts with proteins located in the rat cells endosomal membranes. The expression of the protein’s dedicator of cytokinesis protein 9, synaptosomal-associated protein 29, GTP-binding protein Rab3D, Ras-related protein M-Ras, and exocyst complex component 7 was observed only in mastoparans treated cells. Rho GTPase Cdc 42 and exocyst complex component 7 as components of the Ca^2+^-independent FcRI-mediated exocytosis pathway, synaptosomal associated protein 29, and GTP-binding protein Rab3D as components of the Ca^2+^-dependent FcRI-mediated exocytosis pathway and Ras-related protein M-Ras, a protein that is related to the mediation of cell shaping and proliferation following exocytosis (Delazari dos Santos et al., 2012).

## Interaction With G Proteins

The heterotrimeric guanine nucleotide-binding proteins (G proteins) act as switches that regulate the information processing circuits connecting the cell surface receptors. G proteins are present in all the eukaryotic cells and play important roles as metabolic, humoral, neural, and developmental functions (Simon et al., 1991).

Higashijima et al. showed that mastoparan-L (from *Vespa lewisii*) increases the GTPase activity and the rate of nucleotide binding of several purified G proteins (Higashijima et al., 1988). Mastoparan-L (ML) of 100 micromolars was able to increase the binding rate of guanosine 5'-(3-0-thio) triphosphate (GTPγS) in G_o_ about seven times in the presence of 5 μM Mg^2+^. ML also estimulated GTPase activity of G proteins reconstituted in PE/PC/PS vesicles. At 100 μM Ml, activity of G_o_ and G_i_ were increased by 16 times, and G_t_ and G_s_ were relatively insensitive to ML. The structure of mastoparans plays an important role in G proteins activation: the simple exchange of Lys^12^ to Ala^12^ stimulated G_o_ in comparison to the original peptide (Higashijima et al., 1990).

It was observed that mastoparan-L also stimulates NDPK in similar concentrations to that which stimulates G proteins (Kikkawa et al., 1992). In another work, Klinker et al. reported evidences that mastoparan-L activates G-proteins in HL-60 membranes in an indirect way (through interaction with NDPK) (Klinker et al., 1994). Later, the same group showed that mastoparan increased high-affinity GTP hydrolysis in different cell types as well as NDPK activity in these cells, suggesting that the indirect activation of the G protein by mastoparan-L *via* stimulation of NDPK can be a general mechanism of venom action in cell membranes (Klinker et al., 1996).

## Interaction With Phospholipases

Phospholipases are enzymes that hydrolyze phospholipids, releasing products such as lysophospholipids, fatty acids, and diacylglycerols. These enzymes are classified as phospholipase A_1_, A_2_, C, or D, according to their substrate cleavage site and produce lipid mediators that act in signal transduction in several cell types and tissues, as well as participating in the digestion process (Kikkawa et al., 1992; Tappia and Dhalla, 2014). These enzymes are found in all living organisms; moreover, phospholipases are also found naturally in venoms of various species, including wasps (King et al., 1984; Costa and Palma, 2000; Sukprasert et al., 2013).

Argiolas and Pisano reported that several mastoparans are capable of stimulating the phospholipase A_2_ hydrolysis rate, suggesting that the co-occurrence of these enzymes and mastoparans in the wasp venom can potentiate the deleterious effects during prey envenomation (Argiolas and Pisano, 1983). In addition, one of the main products of the cleavage of phospholipids is arachidonic acid, a precursor of several proinflammatory molecules such as prostaglandins, lipoxins, and thromboxanes (Pruzanski and Vadas, 1991).

Mastoparan-V1 and -V2 from *Vespa vulgaris* associated with phospholipase A_1_ stimulated the release of prostaglandin E_2_ in the mouse macrophage J744 cultures as well as in the mouse peritoneal cells, and synergistic action of these molecules was lethal to mice (King et al., 2003). In 1321N1 cells (human astrocytoma), mastoparan stimulated phospholipase D activity promoting phosphatidylcholine hydrolysis and accumulation of diacylglycerol and phosphatidic acid into the cells. Furthermore, the stimulation of the enzyme does not seem to depend on calcium ions and that this activation does not occur *via* G proteins (Mizuno et al., 1995). In lymphocytic mouse leukemia L1210 cells, pertussis toxin did not influence the activity of phospholipase D stimulated by mastoparan-B. Moreover, this peptide stimulated arachidonic acid release possibly acting in other phospholipases present in cell membranes indicating that the activity does not appear to be mediated *via* G-proteins (Lee et al., 1998). Mastoparan stimulates expressed phospholipase D in cell membranes independently of G_i_, protein kinase C, and calcium in the rat (RBL-2H3) mast cell line (Chahdi et al., 2003). In addition, it was observed that mastoparan was able to stimulate phosphoinositide-specific phospholipase C in human myocardial tissue independently of the activation of G protein (Schnabel et al., 1997).

Interestingly, in contrast to what was previously discussed in this section, there are reports indicating that ML also exhibits inhibitory effects on phospholipases. In human astrocytoma cells (1321Nl), Nakahata et al. showed that ML strongly inhibited GTPγS-induced inositol phosphate accumulation, but not the accumulation induced by Ca^2+^ (Nakahata et al., 1990). The observed inhibitory effect was resistant to treatment with pertussis toxin, suggesting that ML inhibits a G-protein which activates the phospholipase C, which is insensitive for the pertussis toxin. Another report showed that mastoparan-L also interfered in the hydrolysis of inositol phosphate in an electrically permeabilized human neuroblastoma SH-SY5Y cells stimulated with GTPγS (Wojcikiewicz and Nahorski, 1989).

In plants, it was observed that mastoparan-L promotes the activation of phospholipases, acting as an important tool for cell signaling studies. In zucchini and sunflower hypocotyl segments, phospholipase A_2_ was activated by mastoparan-L, releasing phosphatidylcholine and lysophosphatidylcholine (Scherer, 1995). It was also observed that mastoparan activates phospholipase C in carrots (Cho et al., 1995) and soybean cells (Legendre et al., 1993), phospholipase C and D in *Chlamydomonas* (Munnik et al., 1998; Himbergen et al., 1999), and phospholipase D in carnation petals (de Vrije and Munnik, 1997).

## Antimicrobial Activity

Antimicrobial peptides have been attracting attention due to the increasing number of bacterial resistance to the conventional antibiotics (Phoenix et al., 2013; Lei et al., 2019; Zhang et al., 2021). These molecules have several structural motifs, are found in a wide variety of living beings, and play an important role in the innate immunity and defense of these organisms (Epand and Vogel, 1999; Moretta et al., 2021). Like so many other biologically active peptides, mastoparans have been extensively studied for their antimicrobial properties (Table 1). The first work to describe a mastoparan (mastoparan-L) with antibacterial activity was performed by Katsu et al*.* It was reported that this peptide promotes the release of phospholipid and efflux of K^+^ indicating higher toxicity in *S. aureus* and weaker toxicity in *Escherichia coli* and human erythrocytes (Katsu et al., 1990).

Park et al*.* reported that mastoparan-B (MP-B), isolated from *Vespa basalis*, displayed potent antimicrobial activity against Gram-positive and Gram-negative bacteria (MIC values of 3.3 mg mL^−1^ for *Enterococcus faecalis* LS-101 and *Bacillus subtilis* PCI 219 and of 6.25 mg mL^−1^ for *Shigella flexneri* EW-10 and *S. sonnei* EW-33), while mastoparan-L was active only against Gram-positive bacteria (Park et al., 1995). It was later observed that mastoparan-L was effective against some strains of *Brucella abortus* (Halling, 1996).

In another study, mastoparan-L was used as a template for the development of an analog created by the replacement of Ala^5^ and Ala^8^ by Ile and Arg residues, respectively. This peptide analog displayed a potent antimicrobial activity against susceptible and multidrug-resistant bacteria and *Candida* ATCC strains. It did not exhibit cytotoxic activity against rat erythrocytes and human embryonic kidney cells (HEK-293). However, it was cytotoxic to THP-1-derived macrophages (Irazazabal et al., 2016).

MP-B was shown to be effective against several Gram-positive and Gram-negative pathogenic bacteria with low activity against probiotics. The hydrophobicity change of MP-B without the net positive charge resulted in the analog MP-B-1, where the substitution of Leu^3^ by Trp^3^ led to a more hydrophilic profile due to the hydrogen bond supported by the indole ring group. This single substitution analog became more effective than MP-B, and even up to eight times more effective on some of the bacterial species tested (Yang et al., 2013a).

Mastoparan-M peptide displayed a broad-spectrum antimicrobial activity against Gram-positive and Gram-negative bacteria. In addition, it induced a moderate level on mast cell degranulation and hemolysis (Lin et al., 2011). Li et al. reported that an all-D enantiomer of mastoparan-M was twice as potent as compared to the natural all-L form against Gram-positive and Gram-negative bacteria and also more stable against proteases degradation (Li et al., 2000). The mastoparan-like peptides (12a, 12b, 12c, and 12d), found in *Vespa mandarinia*, showed activity against Gram-positive and Gram-negative bacteria and fungi, with a low hemolytic activity (Xu et al., 2006a; Xu et al., 2006b).

Mastoparan-VT1 (from *Vespa tropica*), identical to mastoparan-M from *Vespa mandarinia*, showed a broad-spectrum activity against standard and clinically isolated strains with MIC ranging from 2.5 to 10 μg mL^−1^ to Gram-positive, 5–40 μg mL^−1^ to Gram-negative, and 10–40 μg mL^−1^ against *Candida* strains. This work also described six new peptides named mastoparan-VT2 to mastoparan-VT7 with different antimicrobial activities (Yang et al., 2013b).

The peptide mastoparan-V1 (MP-V1, isolated from *Vespula vulgaris* venom) is an atypical mastoparan with 15 amino acids length and shows a low hydrophobicity with the grand average of hydropathicity (GRAVY) value of - 0.053. MP-V1 showed superior antimicrobial activities compared to mastoparans MP-L, MP-X(V), and MP-B and also a more potent hemolytic activity promoting ∼20% hemolysis on erythrocytes at 100 μM (Kim et al., 2016). Dominulin-A and -B are other unusual mastoparans composed of 17 amino acids found on the cuticle and in the venom of females of the social paper wasp *Polistes dominulus*. Preliminary microbiological assays showed that dominulin MIC values are in the range of 1–8 μg mL^−1^ including these peptides among the best antibacterial peptides isolated to date. Curiously, these peptides were also found in old female cuticle and nest paper, suggesting that dominulins should play an important function to protect the colony from infections (Turillazzi et al., 2006).

A novel mastoparan peptide, named Mastoparan-S, was isolated from the hemolymph of the praying mantis *Sphodromantis viridis,* popularly called the African mantis. Mastoparan-S exhibited a broad antimicrobial activity (Gram-positive and Gram-negative bacteria, and fungi), with a higher antimicrobial activity against Gram-negative bacteria. This peptide showed low hemolytic activity; however, had significant cytotoxicity on the HeLa cells. Interestingly, mastoparan-S presents similarity with the MP-B. Moreover, this is the only mastoparan described so far that does not belong to the order Hymenoptera (Zare-Zardini et al., 2015).

Investigating the venom of *Vespa* species (*V. affinis, V. analis, V. basalis, V. ducalis, V. mandarinia,* and *V. velutina flavitarsus*) in Taiwan, Lin et al*.* cloned six cDNAs encoding the precursor polypeptides of MPs (Lin et al., 2011). Among them, the amino acid sequences of MP-A (Nakajima, 1984), -B (Ho and Hwang, 1991), and-M (Hirai et al., 1981) had been previously reported. MP-AF was isolated from the venom of *V. affinis* by Shyu et al*.* (unpublished data). A total of two novel peptides were identified from the cDNA sequences from *V. ducalis* and *V. velutina*, and named MP-D and -V, respectively (Lin et al., 2011). However, MP-D has the same sequence of the MP-T previously identified by Nakajima (Nakajima, 1986). The peptide named MP-V is identical to mastoparan-X (Hirai et al., 1979a) and mastoparan-like peptide 12a (Xu et al., 2006b). The antimicrobial activities of all the six synthesized peptides were evaluated and they exhibited a broad-spectrum antimicrobial activity against several resistant bacterial strains: *S. aureus* (CCRC 12652, CCRC 15211), *Streptococcus alactolyticus* (clinical isolate), *Staphylococcus xylosus* (clinical isolate), *Salmonella choleraesuis* (clinical isolate), *Salmonella typhimurium* (CCRC 12947), *Escherichia coli* (BL21, JM109 AmpR), *Klebsiella pneumoniae* (CCRC 10694), *Vibrio parahamelytics* (CCRC 10806), *Citrobacter koseri* (clinical isolate), and *Pseudomonas aeruginosa* (clinical isolate). Among the tested bacteria, the lowest MICs or MBCs values were at 1.5–3 μg mL^−1^ against *S. xylosus* where MP-AF showed the best activity. On the other hand, the highest MICs or MBCs values at 96–256 μg mL^−1^ were obtained against *P. aeruginosa*, and again MP-AF exhibited the best performance (Lin et al., 2011).

Mastoparan-AF was assayed against multiple-antibiotic-resistant *E. coli* isolates alone or in combination with six clinically used antibiotics (ampicillin, cephalothin, chloramphenicol, gentamicin, neomycin, and tetracycline). Most of the multidrug-resistant *E. coli* tested were susceptible to MP-AF with MICs values ranging from 4 to 16 μg L^−1^. MP-AF also acts synergistically with the specific antibiotics (*e.g.*, cephalothin or gentamicin) against some resistant *E. coli* isolates (PFL6 and PFH13). Although it was neither synergistic nor antagonistic against the other strains (PFH1 and PFH12) when in combination with each of the six antibiotics (Lin et al., 2012). It was also observed that MP-AF alone or in combination with specific antibiotics exhibited a potent antimicrobial activity. MP-AF acted in synergism with (*e.g.*, ciprofloxacin, trimethoprim/sulfamethoxazole, or colistin against multidrug-resistant *Acinetobacter baumannii)*, a common nosocomial pathogen causing severe infections, especially to the immunocompromised patients in healthcare settings (Lin et al., 2017).

Mastoparan-L was effective against colistin-susceptible and colistin-resistant *A. baumannii* with MIC value at 4 mg mL^−1^ for colistin-susceptible *A. baumannii* and 1 mg mL^−1^ for colistin-resistant *A. baumannii.* MP-L was also tested against 13 colistin-resistant *A. baumannii* clinical isolates, and MP-L exhibited a stronger activity (MIC_50_ 8 mg L^−1^) than colistin (MIC_50_ > 512 mg L^−1^) or indolicidin (MIC_50_ 16 mg mL^−1^) (Vila-Farres et al., 2012). Later, the same group synthesized 10 analogs of MP-L with several structural features including: introduction of D-amino acids, synthesis of retro, enantio, and retroenantio versions of MP-L, modification on both N- (guanidilated or acetylation of the free amine group) or C-terminus (with an extra positive charge by the addition of an ethylamine moiety) of the original mastoparan. Most of these analogs maintained the activity against the colistin-resistant *A. baumannii* clinical isolates, showed moderate toxicity in the HeLa cells (5–13 μM), and exhibited high stability after 24 h in the presence of human serum (Vila-Farrés et al., 2015).

Mastoparan-VB1 (MP-VB1), isolated from *Vespa bicolor* venom, showed a strong antimicrobial activity against bacteria and fungi (*e.g.,* MIC of 3.75 mg mL^−1^ against *S. aureus* ATCC2592; MIC of 120 mg mL^−1^ against *P. aeruginosa* 7A, a clinical-isolated multidrug-resistant strain against methicillin, amoxicillin, ampicillin, cephalothin I, II, III, and IV; and MIC of 15 mg mL^−1^ against *C. albicans* ATCC 2002), and induced mast cell degranulation (about 71% at the concentration of 10 mg mL^−1^). MP-VB1 displayed almost no hemolytic activity on the rabbit and human blood red cells (Chen et al., 2008).

Mastoparans PDD-A and PDD-B isolated from the social wasp *Polistes dorsalis*, PMM from *Polistes major*, MP from *Mischocyttarus phthisicus,* and 40 analogs designed based on these four natural peptides were tested *in vitro* for the assessment of pathogenic bacterial antimicrobial susceptibility, and for the hemolytic and mast cells degranulation activities (Čeřovský et al., 2008).

Mastoparan PDD-A did not differentiate between Gram-negative *E. coli* and Gram-positive *B. subtilis* with MIC of 11.8 and 7.5 µM, respectively. Nonetheless, PDD-B and MP were more active against *B. subtilis* and PMM was 3.5 times more active against *E. coli*. PDD-A, PMM, and PM exhibited no hemolytic activity (50% hemolysis at 80 µM or higher concentration), while PDD-B was slightly more hemolytic (50% hemolysis at 45 µM). All the four mastoparans showed a potent mast cell degranulation activity with EC_50_ ranging from 15 to 26 µM (Čeřovský et al., 2008).

Čeřovský et al*.* also synthesized and evaluated the biological properties of the 40 analogs using the four natural mastoparans described previously as templates. The analog PDD-A1 exhibited an enhancement of charge by the exchange of Glu^8^ by Gln^8^ and resulted in a reduced antimicrobial activity against *B. subtilis*, but no loss of activity against *E. coli*. PDD-A-2 and PDD-A-3 analogs obtained by the exchange of Asn^12^ by Asp^12^ and Asn^2^ by Asp^2^, respectively, were shown to be more active against *B. subtilis* in contrast to the natural PDD-A. However, in PDD-A-4 analog, where the two previously mentioned replacements were made, there was a reduction of the antibacterial activity, mainly against *E. coli*. The replacement of Lys^4^ by Ser^4^ resulted in PDD-A-5 and caused a slight loss of activity against both the bacteria. The analogs PDD-A-6, PDD-A-7, and PDD-A-8 were obtained by replacing Lys^4^, Lys^5^, Lys^9^, and Lys^11^ by Ser and showed a gradual reduction in the antimicrobial activity, whereas PDD-A-8 with all the Lys exchanged by Ser resulted in a total loss of activity. Similar to analog PDD-A-5, the analogs PDD-A-9, PDD-A-10, and PDD-A-11 were produced by replacing separately the other three Lys residues in the peptide by Ser and resulted in the antimicrobial activity of the same order of magnitude. For the analog PDD-A-12, the two amino acids at the N-terminal extremity were rearranged enhancing the hydrophobic moment but resulted in no augment in the antimicrobial activity (Čeřovský et al., 2008).

In PDD-B analogs, the substitution of Lys residues by more basic Arg residues resulted in an increased hemolytic activity and had no effect on the antibacterial activity (PDD-B-2). In PDD-B-4, the replacement of Leu^4^ by Lys^4^ resulted in an increased activity against *E. coli* and a drastic reduction in the hemolytic activity. In PDD-B3, the replacement of Trp^3^ by Phe^3^ resulted in the loss of activity against *E. coli* and on the rat erythrocytes, while the replacement of Trp^3^ by Ser^3^ (PDD-B-5) resulted in a total loss of activity. Analog PDD-B-1 obtained by replacing Leu^14^ by Ile^14^ is active mainly against *B. subtilis*, but also showed a moderate antimicrobial activity against *E. coli* and on the rat erythrocytes (Čeřovský et al., 2008). Interestingly, a mast cell degranulation peptide with the same sequence had been purified from *Polistes rothneyi iwatai* and named Polistes-mastoparan-R1 (Murata et al., 2006).

In general, the MP analogs exhibited a reduced activity in antibacterial assays, mainly against *E. coli*. However, the addition of a Fmoc group at the N-terminal portion of the MP-3 and MP-7 analogs resulted in an increase of the hemolytic activity without changing the antimicrobial potency. When the C-terminal amidation was removed, the analog MP-9 showed a reduction in the antibacterial and hemolytic activity (Čeřovský et al., 2008).

PMM analogs, where step-by-step deletion of amino acids at the N- or C-terminal, showed a gradual decrease in the antimicrobial activity. The analog PMM-12, produced by the inversion of the amino acids Ile^1^ and Asn^2^ in addition to the substitution of Gly^10^ for Ala^10^, exhibited a slight augment of the antimicrobial activity and greatly increased the hemolytic activity (Čeřovský et al., 2008).

The peptide Parapolybia-MP, isolated from *Parapolybia indica* (Nakajima, 1986), exhibited a potent antimicrobial activity against Gram-positive and Gram-negative bacteria with MIC values ranging from 2.9 to 15.6 mg mL^−1^, caused hemolysis with ED_50_ value equal to 3.7 × 10^–5^ M, and no mast cell degranulation activity (de Souza et al., 2011).

Polybia-MP-I, -II, and-III are mastoparans isolated from the social wasp *Polybia paulista*. Polybia-MP-I presented a high chemotaxis of polymorphonucleated leukocyte (PMNL) cells, induced no hemolysis to rat erythrocytes, and caused the lysis of the rat mast cells, leading to the delivery of their granule contents (de Souza et al., 2005). It showed a potent antimicrobial activity against the Gram-positive and Gram-negative bacteria with a similar antimicrobial activity against *E. coli, S. aureus,* and *Staphylococcus epidermidis*, while is more active on *B. subtilis*. Polybia-MP-I caused the outer and inner membrane permeabilization in *E. coli* (Wang et al., 2013). Polybia-MP-II (previously identified as peptide 13a by (de Souza et al., 2004)) presented potent chemotactic activities of rat PMNL cells, induced high hemolysis to rat erythrocytes and promoted degranulation of 57% of the rat peritoneal mast cells at the concentration of 6.2 × 10^–5^ M (de Souza et al., 2004). Regarding Polybia-MP-III, and similar to Polybia-MP-II, this MP caused hemolysis and induced mast cell degranulation through the mast cell membrane disruption (de Souza et al., 2009). Polybia-MP-III had a less potent antimicrobial activity than Polybia-MP-II for the Gram-positive and Gram-negative bacteria. For Polybia-MP-II, the MIC values were in the range of 2–38 μM in contrast to the MIC values in the range of 19–310 μM obtained for Polybia-MP-III (de Souza et al., 2009).

As previously described, Polybia-MPI induced no hemolysis and is highly selective of the bacterial cells. Cabrera et al*.* demonstrated that Polybia-MPI selectivity is higher on the anionic over the zwitterionic vesicles, impaired by the presence of cholesterol or cardiolipin. It was suggested that the selectivity of Polybia-MP-I could be attributed to the presence and position of Asp residues that enable the equilibrium of the electrostatic interactions and favor the preference for the more hydrophilic environment (dos Santos Cabrera et al., 2008).

A Polybia-MPII peptide isolated from the social wasp *Pseudopolybia vespiceps testacea* showed activity against bacteria and fungi. This peptide presents a potent *in vitro* activity against *S. aureus* with ED_90_ of 2.9 µM, and reduced mycobacterial growth by 80% at 12.5 μM in *in vivo* assays using the peritoneal macrophages from BALB/c mice. Antifungal activity against *C. albicans* with ED_90_ of 15.3 µM and for *Cryptococcus neoformans* with ED_90_ of 22.7 µM was detected. Bactericidal activity *in vivo* was also evaluated using 57BL/6 mice in a topical infection model with *S. aureus* and treated with 5 mg kg^−1^ of peptide, showing a significant decline of the bacterial load after six days of topical treatment (Silva et al., 2017).

The emergence of multidrug-resistant (MDR) *tuberculosis* is a major threat to human health worldwide, and the occurrence augment of nontuberculous mycobacterial infections (particularly produced by *Mycobacterium avium*) have created an urgent need to develop new antibiotics. Mastoparan L showed antimicrobial activity against the clinical isolates of *M. tuberculosis* with MIC values ranging from 32 to 64 mg mL^−1^ and MIC >128 μg mL^−1^ for *M. avium* isolates (Portell-Buj et al., 2019).

Eumenine mastoparan-AF (EMP-AF), a mast cell degranulation and hemolytic peptide from *Anterhynchium flavomarginatum micado*, in its natural form (with amidated C-terminus), is more active against Gram-positive and Gram-negative bacteria than its analog with carboxylated C-terminus. In addition, when the first three residues of amino acids from the N-terminal were removed, the resulting analogs showed no activity (Konno et al., 2000; dos Santos Cabrera et al., 2004). A total of two novel eumenine mastoparan EMP-EF and EMP-ER were described from *Eumenes fraterculus* and *E. rubrofemoratus*, respectively. These peptides exhibited a broad-spectrum against microorganisms, low mast cell degranulation, and hemolytic activity (Rangel et al., 2011). Similar results were observed with novel eumenine mastoparans named EMP-EM1 and EMP-EM2, isolated from the wasp *Eumenes micado* (Konno et al., 2019). Eumenine mastoparan-OD (EMP-OD, from *Orancistrocerus drewseni*) which is structurally similar to EMP-AF, however, showed low bactericidal activity, moderate hemolysis, and higher activity against *C. albicans* and *Botrytis cinerea* (Murata et al., 2009; Baek and Lee, 2010).

Due to the high morbidity and high mortality associated with intravascular stent infection, agelaia-MPI and Polybia-MPII were tested against MDR *Acinetobacter baumannii* biofilm on the vascular stents. It was observed that agelaia-MPI and Polybia-MPII inhibited *A. baumannii* biofilm formation. Agelaia-MPI and polyethylene glycol (PEG 400) coating reduced in 90% of the bacterial adherence on the vascular stents, revealing the potential applications of these peptides as antimicrobials to treat the biofilm-resistant agents (das Neves et al., 2019). Recently, another drug delivery system was proposed using a chitosan-encapsulated mastoparan-C nanoconstruct (Mast-Cs NC) against MDR *A. baumannii* clinical isolates*.* Mast-Cs NC displayed a synergistic bactericidal effect through damaging the bacterial cell membrane. Mast-Cs NC also induced a significant reduction in the bacterial count in a mouse sepsis model compared with chitosan and mastoparan alone (Hassan et al., 2021).

Mastoparan-C (MP-C) shows strong antimicrobial and anticancer activities, but also displays a high cytotoxicity toward the normal mammalian cells (Yoon et al., 2015). To potentially ameliorate these side effects, three novel analogs of MP-C were designed named L_1_G, L_7_A, and L_1_GA_5_K. When combined with antibiotics (gentamicin, rifampin, and polymyxin B), these peptides exhibited synergy or additive effects against *E. coli* ATCC 25922 and *P. aeruginosa* ATCC 9027. These new analogs did not exhibit hemolytic activity toward the mouse RBCs or cytotoxicity on HEK293T cells; they showed anti-inflammatory activity where the level of IL-6 was inhibited and the level of IL-10 was increased in the mouse plasma. These analogs were relatively safe considering their *in vivo* acute toxicities. In particular, the analog L_1_GA_5_K exhibited a strong antimicrobial activity against rifampin-resistant *E. coli* (RRE) and high selectivity. It could prevent the emergence of rifampin resistance in *Enterobacter,* but also reverse rifampin resistance in RRE when used in combination with rifampin (Zhu et al., 2021)*.*

A chimeric derivative of a truncated mastoparan sequence and NCR247C (whereas NCR247C corresponds to the 12 amino acid long C-terminal half of NCR247, a nodule-specific cysteine-rich peptide with an antimicrobial activity) significantly increased the bactericidal activity and altered the antimicrobial spectrum with no hemolytic activity or cytotoxicity on the human cells. The properties of NCR derivatives make them promising antimicrobial therapeutic candidates (Jenei et al., 2020).

A total of three mastoparans analogs with four Lys residues in different positions in the peptide sequences and net charge +5 were designed with the additional modifications: in MK4589 when compared with MK5789, Leu^4^ was replaced by Lys^4^ and Lys^7^ was replaced by Ala^7^; in MK58911 when compared with MK5789, Lys^7^ was replaced by Ala^7^ and Ala^11^ by Lys^11^. These analogs presented a broad-spectrum antibacterial activity, with an extremely potent action against Gram-positive and -negative bacteria, anti-*C. albicans* activity, with low cytotoxicity in HaCaT cells, and nonteratogenicity and no developmental disturbances in zebrafish embryos (Galeane et al., 2019).

Until now, only a few mastoparans were tested successfully on protozoa. The eumenime mastoparans EMP-ER, EMP-EF, and EMP-AF showed a moderate activity against promastigotes of *Leishmania major* with an IC_50_ ranging from 20 to 40 µM (Rangel et al., 2011). Recently, this same group showed that EMP-EM1 and EMP-EM2 exhibited leishmanicidal activity with IC_50_ = 36 µM. Mastoparan-X was tested against *Plasmodium berghei* ookinetes, *P. falciparum* gametocytes, and *Anopheles gambiae*, but displayed a low activity when compared to other peptides tested as melittin and duramycin (Carter et al., 2013). Recently, Vinhote et al*.* showed that mastoparan named venom protein 13b, isolated from *Polybia paulista* (de Souza et al., 2004), was effective against all the developmental forms (epimastigotes, trypomastigotes, and amastigotes) of *Trypanosoma cruzi*, promoting alterations on the transmembrane mitochondrial potential and increase on ROS. Moreover, this peptide seems to interact to *Tc*GAPDH (glyceraldehyde-3-phosphate dehydrogenase from *T. cruzi*), an important enzyme of *T. cruzi*’s life cycle, that regulate many biological processes, such as cell death (Vinhote et al., 2017).

Pronectarina-MP, isolated by Dohtsu et al*.* with the mast cell degranulation activity (Dohtsu et al., 1993), also displays activity against bacteria and hemolysis of the rat red blood cells (de Souza et al., 2011). Pronectarina-MP, when C-terminal amidation is replaced by the acidic form (OH), showed a decrease of the antimicrobial activity, as well as hemolytic and mast cell degranulation properties. This replacement also changes the way the peptide interacts with the zwitterionic PC or anionic PCPG vesicles, suggesting a role of amidation in promoting the stabilization of the secondary structure of the peptide, which seems to be a determinant of its activity (da Silva et al., 2014).

An analog of mastoparan-L, named mast-MO, was proposed and displayed the potent immunomodulatory and direct antimicrobial activities. In the animal models, mast-MO induced the recruitment of leukocytes to the infection site, and reduced the proinflammatory factors, such as IL-12, TNF-α, and IL-6. The second generation of analogs based on mast-MO were no longer toxic against the human cells and presented an increased anti-infective activity against the clinically relevant bacteria both *in vitro* and *in vivo* (Silva et al., 2020b).

The mode of action of these peptides against the microorganisms seems to be the same for the other groups of antimicrobial peptides, interacting directly in the cell membrane, forming pores, and this interaction is dependent on the lipid composition of the membrane as well as the physicochemical characteristics of these molecules (Huang, 2000; dos Santos Cabrera et al., 2008).

Recently, the antifungal action against *Cryptococcus neoformans* of an engineered mastoparan peptide with four lysine residues in its sequence (MK58911-NH_2_) was reported. The results demonstrated that MK58911-NH_2_ was able to reduce the number and size of fungal cells in the macrophages, in zebrafish embryos, and also in biofilms. These results open the perspective to use MK58911-NH_2_ as a model for the rational development of the novel antifungal compounds (Singulani et al., 2021).

The biological activity of the antimicrobial peptides is closely related to their chemical structure. Thus, some peptides are extensively studied in order to understand their structure and function relationships. Among the mastoparans addressed in this work, mastoparan-L is the most studied. However, mastoparan-M exhibited a better biological activity (lower MIC and lower hemolytic activity). For the comparative purposes, Table 2 highlights the antimicrobial and hemolytic activity of some peptides from various origins and with different ranges of biological activity. In addition to a potent antimicrobial activity, a peptide drug candidate should exhibit low cytotoxic effects such as hemolysis, for example. The peptides melittin (Sun et al., 2005), indolicidin (Ryge et al., 2004), and hylin-a1 (Castro et al., 2009) have considerable antibacterial activity but are very hemolytic, while mastoparan-L has moderate antibacterial activity and is also very toxic (Irazazabal et al., 2016). Mastoparan-M shows potent antimicrobial effects and does not have significant hemolytic activity even though it is very similar to mastoparan-L (Yang et al., 2013b). Temporin-1DRa has a C-terminal portion similar to mastoparans L and M, however, it has a moderate antimicrobial and hemolytic activity (Urbán et al., 2007). LL-37 is a human peptide with important functions, including antimicrobial effects against Gram-positive, Gram-negative, and fungi, and because it is endogenous, its hemolytic activity was observed only in high concentrations (Gunasekera et al., 2020). Given its good biological properties, clinical trials have been carried out with this molecule. Pexiganan is a magainin 2 analog (maginin 2 was initially isolated from the African clawed frog *Xenopus laevis*). This analog has a broad spectrum of the antimicrobial activity, was tested in phase III clinical trials for the treatment of diabetic foot ulcers, however, its approval was denied by the FDA as it was not more effective than the commercially available drugs (Ge et al., 1999).

**TABLE 2:** Comparison of antimicrobial and hemolytic properties of selected antimicrobial peptides from different sources.

MIC (µM)								
Microorganism	Melittin^a^	Pexiganan^c^	Indolicidin^d^	LL-37^f^	Temporin-1DRa^h^	Hylin-a1^i^	Mastoparan-M^j^	Mastoparan-L^k^
*Escherichia coli* ATCC 25922	6	3.2–6.4	2	0.31	12.5	32	-	25
*Pseudomonas aeruginosa* ATCC 27853	12.5	3.2–6.4	8.3^e^	0.62	25	8	3.4	50
*Enterococcus faecalis* ATCC 29212	6	25.8	—	6.2^g^	25	16	1.7	25
*Staphylococcus aureus* ATCC 25923	0.08^b^	—	2	1.25	—	8	3.4	12.5
*Candida albicans* ATCC 90028	6	—	—	1.25	25	16.7	6.8	—
Hemolysis (HC_50_)	0.6	201	19	>80	70	18.5	—	>10

MIC, minimal inhibitory concentration; HC_50_, peptide concentration that produces 50% hemolysis.

a (Sun et al., 2005);

b (Mahmoudi et al., 2020);

c (Ge et al., 1999);

d (Ryge et al., 2004);

e (Giacometti et al., 1999);

f (Gunasekera et al., 2020);

g (Leszczynska et al., 2013).

h (Urbán et al., 2007);

i (Castro et al., 2009);

j (Yang et al., 2013b);

k (Irazazabal et al., 2016).

## Anticancer Activity

Cancer is one of the most prevalent diseases in the world, and treatments are based on radiotherapy, chemotherapy, hormone therapy, immunotherapy, stem cell transplant, surgery, and target therapy (Urruticoechea et al., 2010; National Cancer Institute, 2021; Sung et al., 2021). Despite the major advances in chemotherapy, these drugs usually have strong side effects and could develop resistance (Nussbaumer et al., 2011; Gonzalez-Fierro and Dueñas-González, 2021). In recent years, several peptides have been tested against the cancer cells (Gaspar et al., 2013), however, only a few studies have reported mastoparans as potential anticancer peptides.

The first study to report that mastoparans exhibit anticancer properties was made by Raynor et al*.* showing that mastoparan-L, mastoparan-X [isolated from *Vespa xanthoptera* (Hirai et al., 1979a)], and mastoparan-J [from *Polistes jokahamae* (Hirai et al., 1980)] were cytotoxic against the leukemia HL60 cells (Raynor et al., 1992). Later, Kamath et al*.* showed that mastoparan-L inhibited cell invasion and migration of MDA-MB-231 cells and potently inhibited all the migration of MCF7 cells (Kamath et al., 2001). Based on the report by Pfeiffer et al*.* where it was observed that ML is a potent facilitator of the mitochondrial permeability transition (Pfeiffer et al., 1995), Yamada et al*.* developed a transferrin-modified liposomes directed to cells exhibiting super-expressed transferrin receptors stimulating the uptake of liposomes by endocytosis. Thus, using the human chronic myelogenous leukemia K562 cells were observed that, after internalization, the endosome was degraded and ML was successfully released into the cytosol and attacked the mitochondria, inducing the formation of pores and the consequent release of cytochrome C and cell death (Yamada et al., 2005). It is now well established that mastoparans are capable of triggering the mitochondrial apoptosis (Colella et al., 2021).

ML induced the cell death *in vitro* with different levels of cytotoxicity in several cancer cell lines. It was observed that ML exhibited an IC_50_ = 77 µM to Jurkat cells (acute T cell leukemia) and 432 µM to MCF-7, while the nontumor cells (melan-a and HaCaT) were more resistant (IC_50_ values were 411.5 and 428 μM, respectively) (de Azevedo et al., 2015). ML-treatment of B16F10-Nex2 (murine melanoma) cells induced effects such as translocation of phosphatidylserine, DNA fragmentation, activation of caspases-9, -12, and -3, mitochondrial membrane depolarization, an increase of cytochrome C release, generation of reactive oxygen species (ROS), upregulation of the proapoptotic Bax and Bim, and downregulation of antiapoptotic Bcl-XL proteins (de Azevedo et al., 2015). Mastoparan-L reduced the tumor volume and prolonged the mice survival rate in a syngeneic murine melanoma model, without any weight loss or any other evident toxic effect (de Azevedo et al., 2015).

Hilchie et al. showed that mastoparan-L is effective against a large number of cancer cells lines including Paclitaxel-resistant P-glycoprotein-overexpressing MCF7-TX400 cells (Hilchie et al., 2016). This peptide was more toxic to several lines of cancer cells (such as Jurkat T-ALL cells and MDA-MB-231 human breast cancer cells) than to the nontumoral cells (PBMCs and HMECs) and devoided hemolytic activity. It was observed that ML is a direct-acting anticancer peptide in both the cancer cell lines. Synergistic models were employed to evaluate the effects of ML in combination with anticancer conventional drugs *in vitro* (etoposide, Jurkat T-ALL cells) and *in vivo* (gemcitabine, against a mouse model of mammary carcinoma). The data suggest that ML may be able to potentiate the cytotoxic effects of these drugs (Hilchie et al., 2016).

Polybia-MPI, which was initially described as a mast cell lytic, chemotactic, and antibacterial peptide (de Souza et al., 2005), is cytotoxic against the prostate and bladder cancer cells (PC-3, Biu87, and EJ) and also for the umbilical vein endothelial cell line HUVEC with IC_50_ value ranging between 25 and 36 µM in the LDH assay and 55–64 µM in the MTT assay. However, the peptide showed low activity against the nontumorigenic murine fibroblast cell line NIH3T3 (Wang et al., 2008). Considering the importance of the structure–activity relationships, three analogs were proposed with the substitution of Leu^7^, Ala^8^, or Asp^9^ by L-Pro in the peptide Polybia-MPI. The L-Pro substitution reduced or promoted the disruption of the α-helix conformation, leading to a reduction of the antitumor activity (Wang et al., 2008). Another work performed by the same group showed that the C-terminal amide substitution by a thioamide (CS-NH_2_) in a Polybia-MPI analog was able to enhance the activity against the cancer cells *in vitro*. In addition, this analog was six-fold less toxic in a murine model *in vivo,* although showed a slight increase in the hemolytic activity. This analog significantly decreased the tumor size in a mouse xenograft sarcoma when compared to the natural form of the peptide (Zhang et al., 2010).

In leukemic cells (K562 and its multi-drug resistant subline K562/ADM), Polybia-MPI showed similar antiproliferative effects with IC_50_ of 26 µM. In comparison, the K562/ADM cells were 201 times more resistant to Adriamycin^®^ and 14 times to Actinomycin D^®^ (Wang et al., 2009). In these cells, Polybia-MPI promotes a rapid rupture of the cell membrane or forming transmembrane pores, suggesting cell death by necrosis (Wang et al., 2009). Polybia-MPI also shows selectivity to Jurkat cells, a human leukemic T-lymphocyte cell line, when compared to the human primary lymphocytes, promoting cell lysis and inducing necrosis but not causing DNA fragmentation. Furthermore, this peptide showed the highest pore-forming activity in the bilayers composed of phosphatidylcholine or a mixture of phosphatidylcholine and phosphatidylserine, while lowering its activity in the presence of membranes containing cholesterol or cardiolipin (dos Santos Cabrera et al., 2012). Against glioblastoma multiforme (T98G cell line), an aggressive brain tumor, the peptides Polybia-MPI, mastoparan-X, and HR-1 (isolated from *Vespa orientalis*) showed cytotoxicity in a dose-dependent manner and promotes alterations in cell morphology with the shrinkage and loss of cell prolongations and nuclear condensation. T98G cells treated with 20 µM of these peptides showed a low annexin V-FITC labeling and little alterations on the membrane mitochondrial potential, while for propidium iodide and intracellular resting calcium there was an expressive labeling, indicating a direct action of peptides in the cell membrane resulting in necrosis (da Silva et al., 2018).

The mastoparan-A from *Vespa analis* and mastoparan-C from *V. crabro* were tested against SK-OV-3 and NIH:OVCAR3 (ovarian tumor cells). Mastoparan-A was more effective against the tumor cells, resulting in an important decrease of cell viability when treated with 100 μM, while mastoparan-C was less effective (Yoon et al., 2015). Later, mastoparan-C and analogs were tested against the nonsmall cell lung cancer H157, melanocyte MDA-MB-435S, human prostate carcinoma PC-3, human glioblastoma astrocytoma U251MG, human breast cancer MCF-7, and normal human microvessel endothelial cells HMEC and showed an increased activity against the cancerous strains being more effective against the PC-3 prostate cancer cells with IC_50_ = 6.29 µM and less active against the noncancer HMEC-1 cell line with an IC_50_ = 57.15 µM. Moreover, these peptides showed low antimicrobial and hemolytic (in human red blood cells) activities. Interestingly, the mastoparan-C showed a strong hemolytic activity in guinea pig and horse erythrocytes (Chen et al., 2018). Mastoparan-M, isolated from *Vespa mandarinia* (Hirai et al., 1981) and also found in other species as *Vespa tropica* [named Mastoparan-VT1 (Yang et al., 2013b)] and *Vespa magnifica* [named mastoparan-like peptide 12c (Xu et al., 2006b)], was used as a template for the design of an all-D peptide that showed an increased cytotoxic activity against the Colo 225, HeLa, and Hep-2 cell lines in comparison to the all-L mastoparan-M version (Wu and Li, 1999).

Li et al*.* applied, for the first time, a fluorinated polyethylenimine (F-PEI) polymer for the transmucosal delivery of Polybia-MPI for the local treatment of orthotopic bladder tumors in mice by the intravesical instillation therapy. MPI/F-PEI displayed improved therapeutic effects compared to mitomycin C, the most commonly used chemotherapeutic agent*,* inhibiting tumor growth and prolonging the mouse lifetime (Li et al., 2019).

In addition to the use as anticancer drug, some mastoparans may also be used as a tool to build drug delivery systems in cancer therapy. Thus, transferrin-modified liposomes carrying pH-sensitive fusogenic mastoparans may be used to deliver the chemotherapeutical drugs into the cytosol of sick cells for cancer therapy (Hoffman et al., 2004).

## Others Activities

### Antiviral Activity

To date, the antiviral activity of mastoparan has been poorly explored. The analogous peptide MAS-7, an analog obtained from mastoparan-L by replacing a Lys^12^ by Ala^12^ and Ile^13^ by Lys^13^, showed to be effective in inactivating several enveloped viruses, presenting reduction of the viral titers higher than 99% in several families as vesicular stomatitis virus (VSV) (Rhabdoviridae), West Nile virus strain NY99 (Flaviviridae), and *Herpes simplex* virus 1 (Herpesviridae), but did not show the activity on adenovirus. In addition, the authors showed that peptide acts directly on the VSV virions promoting their rupture (Sample et al., 2013).

### Activity in Neutrophils

In the human neutrophils, it was observed that mastoparan promotes rapid polymerization of F-actin, followed by the time-dependent depolarization at higher doses in addition to stimulating the rapid formation of phosphatidylinositol 3,4,5-trisphosphate (PIP_3_) and the consumption of phosphatidylinositol 4,5-bisphosphate (PIP_2_), superoxide anion production and upregulation of the complement receptor type 3. However, when cells were treated with pertussis toxin, most of the mentioned effects were blocked, suggesting that these activities may be the result of the activation of G proteins or related proteins (Norgauer et al., 1992). In the mouse macrophage cells, mastoparan-M stimulated the production of TNF-α, IL-1β, and nitrite. The mRNA levels of TNF-α were increased indicating that mastoparan is able to induce an immune response and the recruitment of defense cells (Wu et al., 1999).

### Others Effects of Mastoparans

Mastoparan-B, besides the activities mentioned previously, also promotes the release of histamine from mast cells and induces edema in rats (Ho and Hwang, 1991). In addition, it was observed that peptide and its analogs also cause important cardiovascular changes in rats when applied intravenously.

Ho et al*.* showed that mastoparan-B promotes a potent decrease in blood pressure, and a reduction of the cardiac output and heart rate (Ho et al., 1994). Later, this same group showed that the use of all-D enantiomer of mastoparan-B increases the hypotensive activity of this peptide. The substitution of L-Lys^11,12^ by D-Lys residues improves hypotensive activity and substantially decreases hemolytic activity as compared with the native peptide (Ho et al., 2001).

Studies with mastoparan Polybia-MPII have shown myotoxic activity in mice. When injected intramuscularly, this peptide promoted inflammation and tissue damage, as well as induction of apoptosis and necrosis of muscle fibers (Rocha et al., 2007; Rocha et al., 2008). Subsequently, it was reported that the peptide promotes the expression of caspases-3 and -9, induces significant changes in the structure of the mitochondria, and activates of cytokines, indicating that peptide may play an important role during wasp poisoning (Rocha et al., 2010). Interestingly, the mastoparan named agelaia-MPI (from *Parachartergus fraternus*) with a similar structure to Polybia-MPII possesses antinociceptive effects in mice when injected *via* intracerebroventricular, possibly acting on ionic sodium channels (Gonçalves et al., 2016). However, Baptista-Saidemberg et al. showed that in the pancreatic islets of mice the insulin release induced by the peptide does not interfere in the function of ion channels K^+^_ATP_ and L-type Ca^2+^ (Baptista-Saidemberg et al., 2012). In addition, this peptide promotes a potent degranulation in the rat peritoneal mast cells, hemolytic activity, and has no antimicrobial and chemotactic activity (Mendes et al., 2004).

As mentioned earlier, studies have shown that mast cells play important roles in the adaptive immune system contribution, for example, in the mobilization of neutrophils to the site of bacterial infections, favoring their resolution. Furthermore, the use of the mast cell activators appears to be an interesting class of potent vaccine adjuvants that induce long-term humoral responses (McLachlan et al., 2008; Arifuzzaman et al., 2019). In this direction, mastoparan-7, an analog of the peptide mastoparan-L, was used as adjuvant to produce an anticocaine vaccine to induce an immune response that could attenuate the effects of cocaine usage in humans. Cocaine is poorly immunogenic, so the use of adjuvants is necessary to boost the immune responses against the vaccine antigens. St. John et al*.* described a novel cocaine vaccine formulation using this peptide as a mucosal adjuvant. This formulation induced the production of high quality neutralizing antibodies and reduced cocaine penetration of the blood brain barrier and protected mice from its psychoactive effects (St. John et al., 2020).

The black-bellied hornet *Vespa basalis* is the most dangerous species of vespine wasps found in Taiwan and its venom is highly toxic and painful. It was observed that five venom components (1, 3, 4, 9, and 12) could mimic the venom-induced pain responses in rats. Components 3 and 4 were identified as mastoparan-B and HP-1, respectively, and component 9 was speculated as a novel variant of HP-1/2 (Zhou et al., 2020).

Mastoparan-L acts as a stimulator of monoamine exocytosis and displays anxiolytic-like effects. The anxiolytic-like effect of ML was attenuated by flumazenil and WAY100635 pretreatments. ML reduced the plasma corticosterone and lowered the scoring function at GABAA, 5-HT1A, corticotropin-releasing factor receptor subtype 1 (CRF1), and glucocorticoid receptors (GR) suggesting the involvement of GABAergic, serotonergic, and glucocorticoid mechanisms in its anxiolytic-like property (Silva et al., 2020a).

## Challenges and Limitations for Therapeutic Applications

Although promising, peptide-based drugs pose a challenge for clinical use. Barriers such as selectivity, bioavailability, stability, and resistance to proteolysis need to be overcome. As mentioned earlier, mastoparans are immunogenic and have been tested as an adjuvant in the experimental vaccine models for intranasal *in vivo* applications (St. John et al., 2020).

On the other hand, applications for the other biological activities of mastoparans are still in the early stages. In addition to the desired biological activity, some mastoparans are potentially toxic and have high hemolytic rates, for example, and modifications to these molecules are necessary to eliminate the unwanted effects. The rational design of peptides produced manually or with the aid of software is an interesting strategy to overcome this problem. The addition of the FLPII pentapeptide motif naturally found in peptides from amphibians and some wasp species at the N-terminal extremity of mastoparan-L potentiated the antimicrobial and immunomodulatory effects while reducing the hemolytic activity (Silva et al., 2020b). In another approach, increasing charge, hydrophobic moment, and reducing hydrophobicity in mastoparan-L analogs potentiated the antibacterial activity and significantly reduced the effects against eukaryotic cell lines and hemolytic activity (Oshiro et al., 2019). If, on the one hand, modifications using L-amino acids can improve the desired biological effects and reduce the adverse effects, these molecules are still vulnerable to the action of proteases in a possible oral application. To circumvent this problem, the addition of D-amino acids, proteinogenic amino acids, and other peptidomimetics approaches may be an alternative to be considered (Rezende et al., 2021). Nanotechnology-based systems also represent a promising alternative to prevent the proteolytic degradation of drugs, as well as to potentiate the desired effects. The nanoformulation containing mastoparan and chitosan is effective in killing clinical isolates of multidrug-resistant *Acinetobacter baumannii* and has no hemolytic activity (Hassan et al., 2021).

Another major challenge for the application of peptides in the treatment of diseases of the central nervous system is to cross the blood–brain barrier by noninvasive means. The use of cell-penetrating peptides (CPPs) such as TAT and penetratin has been successfully employed for the transport of various types of molecules, including peptides and proteins, both *in vitro* and *in vivo* (Sharma et al., 2014; Kristensen et al., 2016). Mastoparan-L was tested by conjugating to a transferrin–mastoparan liposome complex. However, due to their high toxicity against erythrocytes and cytotoxicity, as well as the activity on liposomes, they resulted in a lower availability for penetration into the brain when compared to peptides with lower toxicity (Sharma et al., 2014).

## Conclusion

Wasp venom is rich in bioactive substances such as peptides and proteins that exhibit various biological activities of medical interest. Among these compounds, the mastoparans stand out, initially identified by inducing exocytosis of several mammalian cells, the mast cell degranulation being the iconic feature of this group of molecules. Over time, it has been observed that mastoparans present several other important biological properties, highlighting the high number of publications reporting their antimicrobial activities mainly due to the antibiotic crisis and the increase in antimicrobial resistance and the growing interest in these molecules as anticancer agents. On the other hand, while some mastoparans such as mastoparan-L and Polybia-MPI were extensively studied, others such as Protopolybia-MP were poorly investigated. Furthermore, the broad spectrum of activities of these peptides shows that these molecules have the potential to serve as templates for the development of new drugs for several therapeutic applications. All these features justify the crescent interest in mastoparans as therapeutic agents.
